# Multi-Domain Feature Analysis and Application Research of GPR Aliased Signals

**DOI:** 10.3390/s25092741

**Published:** 2025-04-26

**Authors:** Chuan Li, Yawei Wang, Qibing Ma, Xiaorong Wan

**Affiliations:** 1 Faculty of Information Engineering and Automation, Kunming University of Science and Technology, Kunming 650051, China; lichuan@kust.edu.cn (C.L.); wyww1227@163.com (Y.W.); 20222204135@stu.kust.edu.cn (Q.M.); 2 Yunnan Key Laboratory of Computer Technology Applications, Kunming University of Science and Technology, Kunming 650051, China

**Keywords:** aliased signals, multi-domain features, Hilbert transform, STFT, signal processing

## Abstract

In radar detection of concrete structures, the significant differences in electromagnetic properties between rebar and concrete result in strong reflections at rebar interfaces. The electromagnetic waves reflected by the dual-layer rebar interfere with and superimpose, severely obscuring their characteristic signals, making accurate identification challenging. This study investigates the aliasing effect in ground-penetrating radar (GPR) signals through simulation analysis of aliased signals at different rebar spacings and examines their characteristics across the time, frequency, and time–frequency domains. Experimental results indicate amplitude increases in the time domain. The Hilbert transform effectively extracts instantaneous phase inversions, and STFT provides an intuitive time–frequency distribution, facilitating the extraction and analysis of signal features. Additionally, this study includes the design and implementation of an aliasing peak point extraction algorithm with a relative error of less than 10% in practical applications.

## 1. Introduction

During the detection process, the ground-penetrating radar (GPR) sensor receives overlapping and interwoven echo signals, resulting in complex aliasing effects. In the detection of double-layer rebar within concrete structures, multiple reflections from different rebar layers and surrounding media interfere with the acquired signals, obscuring the true characteristics of the target rebar. This aliasing phenomenon significantly complicates the precise identification of rebar positions and the assessment of their structural attributes. If sensor data processing is inadequate, it may lead to misinterpretations of subsurface structural conditions, potentially compromising engineering safety.

Ground-penetrating radar (GPR) is a technology that utilizes high-frequency electromagnetic waves to detect subsurface electrical property variations [1]. Its advantages, including high precision, efficiency, continuous non-destructive detection, real-time imaging, and intuitive results [2,3,4], make it one of the most effective non-destructive methods for tunnel inspection in remote and engineering applications.

In 2024, Fang et al. [5] proposed a GPR-based drainage pipeline inspection robot, integrating MEMS-IMU, encoders, and ultrasonic ranging to enable axial and circumferential scanning. The introduction of the GPR cross-line fusion (CLF-GPR) method significantly enhanced the spatial localization accuracy of external defects. Experimental validation demonstrated axial errors of <2.0 cm, angular errors of <2°, and depth errors of <2.3 cm, highlighting its potential in the inspection of drainage pipelines and the digitalization of urban underground networks. In 2025, Fiorentini et al. [6] explored various approaches, including novel deep learning algorithms for road inventory, advanced methods for pavement crack detection, AI-enhanced GPR imaging for subsurface assessment, high-resolution optical satellite imagery for unpaved road evaluation, and aerial orthophotography for road mapping. Furthermore, transfer learning was employed to apply PyViTENet to GPR measurement data, reconstructing the dielectric constant, shape, and position of scatterers in real-world scenarios. The proposed model exhibited strong generalization capability and accuracy, providing a foundation for non-destructive testing of underground scatterers and their surrounding media [7]. Additionally, complementary information from conventional and locally mapped GPR images was integrated. The fused images preserved the traditional amplitude distribution while enhancing the visibility of weak features, minimizing the risk of experts overlooking critical information [8].

In the time-domain analysis, Alani et al. (2018) conducted a comparative study of ground-penetrating radar (GPR) data at 900 MHz and 2 GHz, revealing that high-frequency GPR enables more precise identification of hyperbolic reflection characteristics associated with double-layer rebar structures in concrete [9]. Subsequently, Sudakova et al. (2019) further substantiated the critical role of frequency selection in detection accuracy, demonstrating that low-frequency antennas (300 MHz) are ineffective in resolving deep interface reflections in peat and swamp permafrost due to severe electromagnetic wave attenuation. From a temporal perspective, increasing the antenna frequency enhances the resolution of received signals, thereby refining detection capabilities [10].

Advancements in rebar detection methodologies have also been noteworthy. Tian et al. (2018) [11] and Tosti et al. (2020) [12] introduced detection techniques incorporating two-dimensional migration, adaptive thresholding, and feature parameter computation, significantly improving the recognition of double-layer rebar structures. Xiang et al. (2020) [13] further refined rebar localization by optimizing amplitude characteristics of reflection waves to extract key feature points (POI) with greater precision. More recently, Li et al. (2023) [14] leveraged peak phase differences and adjacent amplitude variations to enhance the accuracy of signal reconstruction in single-layer structures, further expanding the application potential of GPR technology.

The analysis of data received by GPR sensors has seen significant advancements in phase processing and matching pursuit methods. Time–frequency techniques enable the characterization and identification of signal features across both temporal and spectral dimensions, effectively extracting the time-varying characteristics of non-stationary signals and enhancing signal resolution. Urs Böniger et al. [15] proposed a high-resolution time–frequency analysis method incorporating a phase-shift-aware alternative approach. This method was validated using synthetic data and benchmarked against conventional decomposition techniques, demonstrating superior localization capabilities in both time and frequency domains. In 2015, Harkat et al. [16] investigated propagation time within cavities by measuring the interval between two highly attenuated transitions. They compared three time–frequency methods to provide decision-making guidance for radar signal analysis while circumventing the complexities of distinguishing attenuation effects in dispersive media. Leucei G et al. [17] applied time–frequency analysis to assess defect signals in historic buildings, evaluating both surface and subsurface structural damage as well as moisture distribution. In 2017, Liu et al. [18] utilized short-time Fourier transform (STFT) to analyze the time–frequency characteristics of composite reflection waves in layered air gaps of road surfaces, demonstrating that an overall increase in peak instantaneous frequency effectively indicates stratification. Zhang (2017) [19] utilized F-K filtering in seismic data processing to preserve seismic phase alignment while suppressing mixed noise. By integrating median filtering and iterative curve-threshold denoising, they achieved a clear separation of single-source signals with minimal information loss. In 2020, Wu et al. [20] further applied F-K filtering to denoise GPR data. By computing the F-K spectrum of forward noise records, they preprocessed radar data to effectively suppress noise while enhancing the clarity of reflected signals. Guo Shili et al. [21] studied how the GPR early-time signal (ETS) amplitude changes with the top, width, and bottom of cracks. A “∨”-shaped drop at the crack top helps locate its position, while a “∧”-shaped drop at the bottom aids in identifying the crack type. The width of the crack correlates with amplitude variations, enabling quantitative estimation. The connection between “∨” and “∧” points indicates the direction of the crack, supporting precise and minimally invasive road repair strategies. Kan et al. (2022) [22] proposed a supervised learning method, incorporating the Fourier transform, low-frequency filtering, and Gaussian filtering. By integrating intelligent recognition models with enhanced GPR imaging, their approach enables precise detection of internal voids within road structures, followed by engineering validation to confirm its effectiveness. For enhanced visualization and interpretation of complex GPR data, recent studies have leveraged dual-polarization GPR systems to generate high-resolution 3D images of reinforced concrete [23]. For instance, in a 1 m × 1 m reinforced concrete slab experiment, the dual-polarization system demonstrated its ability to provide an accurate and intuitive representation of internal features such as bidirectional reinforcements, steel bar debonding, and concrete delamination, all from a single scanning direction. The combined use of different polarizations proved particularly effective in revealing comprehensive internal structures.

Traditional signal processing methods, such as f-k filtering [19] and LRSD (low-rank and sparse decomposition), have been widely used for denoising and feature extraction in GPR signals [4]. However, these approaches face limitations when dealing with non-uniform rebar clutter and deep-layer signals. In complex concrete media, traditional methods often struggle to effectively separate signals from rebars located at different depths and positions. Moreover, these techniques tend to lack adaptability to varying material conditions, making it difficult to accommodate structural and environmental variations, which limits their effectiveness in practical engineering applications.

In recent years, machine learning-based methods have attracted increasing attention in GPR signal processing. By learning signal features from training data, these methods offer enhanced generalization capabilities in signal analysis [24]. Nevertheless, machine learning approaches still face challenges in real-time processing, data labeling, and generalization. In particular, in the context of rebar detection, addressing the diversity and complexity of signals under varying concrete structures remains an open issue that warrants further investigation.

This paper presents a method for identifying single-row and double-row steel bars based on multiple signal processing techniques, which overcomes the limitations of traditional single-approach methods. Experiments have verified the effectiveness of the short-time Fourier transform (STFT), Hilbert transform, and normalization analysis in the identification of single-row and double-row steel bars. This provides a new technical solution for the application of ground-penetrating radar in the inspection of complex concrete structures.

Specifically, STFT is employed to extract the time–frequency characteristics of signals, revealing distinct patterns of single- and double-layered rebar in the time–frequency domain. The Hilbert transform is utilized to obtain signal envelopes and analyze phase differences. Additionally, the sensor sets a threshold for normalized amplitude, and when the detected signal exceeds this predefined threshold, the presence of double-layered rebar reflections can be inferred. Experimental results demonstrate significant differences between single- and double-layered rebar in terms of time–frequency characteristics, envelope morphology, cross-correlation functions, and power spectra. These features provide a robust foundation for identifying aliased signals.

## 2. Formation of Reflection Superposition Characteristics and the Principle of Rebar Identification

### 2.1. Principles of Electromagnetic Wave Propagation

**Figure 1:**
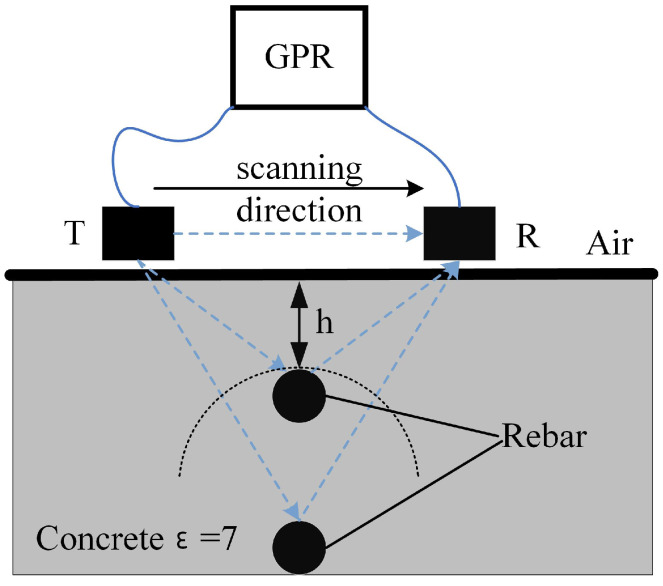
Principle of ground-penetrating radar detection of rebar in concrete.

The propagation of electromagnetic waves is closely linked to the signal detection capabilities of ground-penetrating radar (GPR) sensors [25,26,27]. As an efficient non-destructive testing (NDT) tool, GPR operates by emitting electromagnetic waves and capturing their reflected signals. The antenna sensors serve as the core components of GPR, responsible for both transmission and reception. The transmitting antenna converts electrical signals into electromagnetic waves, directing them into the subsurface, while the receiving antenna detects the reflected signals from underground structures. The core components of ground-penetrating radar (GPR) are the transmitting (T) and receiving (R) antenna sensors, as illustrated in Figure 1. The intensity of the received echo signals is influenced by attenuation, interference, and multipath propagation effects. The signal processing system analyzes the amplitude, phase, and time–frequency characteristics of the echoes to extract critical information regarding the position and depth of target objects, such as reinforcing bars.

The superposition effect of electromagnetic waves within a medium significantly influences the data acquired by sensors. Due to multiple reflections from double-layered reinforcement bars, the superposition of electromagnetic waves is not merely a simple signal summation but also involves coherent interference effects. In GPR surveys, the echo signal received at each detection point is referred to as an A-scan, while multiple consecutive A-scans along a survey line form a B-scan. B-scan data are processed in various ways and visualized as grayscale or pseudo-color images, providing a vertical subsurface profile representation. As electromagnetic waves propagate through the concrete lining medium, their behavior adheres to Maxwell’s equations, which describe the interaction between electric and magnetic fields and their propagation within the medium. Under ideal conditions, the transmission of electromagnetic waves can be expressed as follows:

(1) $$\vec{E}\left(t,\vec{r}\right)={\vec{E}}_{0}\;e^{i(\vec{k}\cdot \vec{r}-\omega t)}$$

In the equation, $\vec{E}\left(t,\vec{r}\right)$ represents the electric field vector, $E_{0}$ is the initial electric field, $\vec{k}$ is the wave vector, ω is the angular frequency, $\vec{r}$ is the spatial position, and *t* represents time. When an electromagnetic wave encounters different media, part of its energy is reflected, while another part is refracted or scattered. The reflection coefficient *R* and the transmission coefficient *T* can be given by the following equations:

(2) $$R=\frac{E_{\mathrm{reflected}}}{E_{\mathrm{incident}}}=\frac{Z_{2}-Z_{1}}{Z_{2}+Z_{1}}$$

(3) $$T=\frac{E_{\mathrm{transmitted}}}{E_{\mathrm{incident}}}=\frac{2Z_{2}}{Z_{2}+Z_{1}}$$

In this equation, $Z_{1}$ and $Z_{2}$ represent the wave impedances of two different media, respectively. To better understand the manifestation of electromagnetic wave superposition effects in double-layer rebar structures, it is essential to first clarify the concept of multipath propagation. In practical applications, radar signals frequently encounter reflective surfaces or object boundaries, leading to multiple reflections. As signals travel through various reflection paths, they superimpose and form complex signal patterns. This multipath reflection introduces phase differences and propagation delays, ultimately resulting in interference effects.

The phase variations primarily arise from changes in the Fresnel reflection coefficient. When the dielectric constants of two adjacent media differ, the phase of the electromagnetic wave in the time domain is altered. The reflection coefficient serves as a key parameter in revealing the complexity of single- and double-layer media signals, as well as the impact of multipath reflection effects. In practical applications, the reflection coefficient can be approximated as follows:

(4) $$\gamma =\frac{\sqrt{\varepsilon_{1}}-\sqrt{\varepsilon_{2}}}{\sqrt{\varepsilon_{1}}+\sqrt{\varepsilon_{2}}}$$

In this equation, $\varepsilon_{1}$ and $\varepsilon_{2}$ represent the relative permittivities of the media on either side of the interface.

### 2.2. Principle of Aliasing Effect

When radar waves encounter double-layer rebar, multiple reflections and scatterings occur, with each reflection introducing different propagation delays. This results in the radar waves traveling along distinct paths within different reflection layers. According to the principle of wave superposition, signals returning from various paths combine based on their respective phase relationships.

If the reflected signals have similar or identical phases, their superposition leads to signal reinforcement, which manifests as peak enhancement in cross-correlation analysis. Conversely, if the phase differences are significant, superposition may result in partial or complete cancellation, thereby reducing signal intensity. This phenomenon is particularly pronounced in the reception of radar signals, where the complexity of multilayer reflections in double-layer rebar significantly influences signal strength and waveform characteristics, ultimately affecting the accuracy of target identification and positioning.

According to wave theory, when two or more electromagnetic waves intersect, the resulting total electric field intensity is the algebraic sum of the individual field intensities. This principle is fundamental in ground-penetrating radar (GPR), as target structures such as rebars and tunnel walls induce multiple reflections and refractions of electromagnetic waves. When these waves interact, the resultant electric (or magnetic) field is the vector sum of the individual wave fields, ultimately leading to variations in signal amplitude. For the electric field, the aliasing principle [28] can be expressed as follows:

(5) $$E_{\mathrm{total}}=\sum_{i=1}^{n}E_{i}$$

In this equation, $E_{i}$ represents the electric field of the ith wave. During electromagnetic wave propagation, the total electric field at any given point is the vector sum of the individual wave fields.

Assuming that the incident electric field of the electromagnetic wave is denoted as $E_{\mathrm{incident}}$ and the reflected electric field as $E_{\mathrm{reflected}}$, the total electric field resulting from the superposition of the reflected wave and the original incident wave can be expressed as follows:

(6) $$E_{\mathrm{total}}=E_{\mathrm{incident}}+E_{\mathrm{reflected}}$$

When analyzing the interference and superposition between incident and reflected waves, it is essential to consider their phase differences. Suppose the reflected wave exhibits a phase difference Δϕ relative to the incident wave; the electric field of the reflected wave can then be expressed mathematically as follows:

(7) $$E_{\mathrm{reflected}}=E_{0}\cos\left(\omega t+\Delta \phi \right)$$

In this expression, $E_{0}$ denotes the amplitude of the reflected wave, ω is the angular frequency of the electromagnetic wave, *t* represents time, and Δϕ indicates the phase difference between the incident and reflected waves. This phase difference can be further defined mathematically as follows:

(8) $$\Delta \phi =\frac{2\pi }{\lambda }\times 2d$$

Here, λ represents the wavelength of the electromagnetic wave propagating within the medium, measured in centimeters (cm) or meters (m). Specifically, for a 400 MHz radar wave propagating through concrete, the wavelength λ is approximately 26.5 cm. Meanwhile, *d* denotes the spacing between the two layers of rebar, expressed in centimeters (cm) or meters (m). Consequently, the total electric field $E_{\mathrm{total}}$ can be represented as follows:

(9) $$E_{\mathrm{total}}=E_{0}\cos\left(\omega t\right)+E_{0}\cos\left(\omega t+\Delta \phi \right)$$

By applying trigonometric identities, the expression for the total electric field $E_{\mathrm{total}}$ can be expanded into the following form:

(10) $$E_{\mathrm{total}}=2E_{0}\cos\left(\frac{\Delta \phi }{2}\right)\cos\left(\omega t+\frac{\Delta \phi }{2}\right)$$

Depending on the direction of reflection and phase difference, reflected waves can either enhance or diminish the resultant wave amplitude. Specifically, when incident and reflected waves are in phase (Δϕ=0,2π), constructive interference occurs, increasing the electric field intensity through superposition. Conversely, when the waves are out of phase (Δϕ=π,3π), destructive interference arises, resulting in reduced or weakened electric field strength.

**Figure 2:**
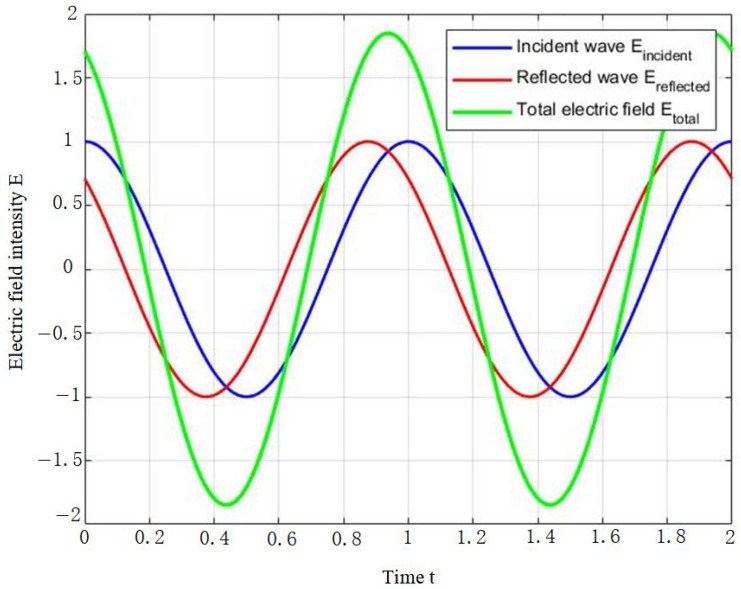
Illustration of electromagnetic wave interference and superposition.

The phase differences between different reflective layers cause signals traveling along various paths to overlap at different time delays, resulting in distinct interference effects. In the case of double-layer rebar, particularly in materials with strong reflective properties such as metal or reinforced steel, radar waves may undergo multiple reflection paths after being reflected, leading to varying signal delays. In this scenario, the superposition of signals is not merely a simple summation of their intensities but also involves phase interference, which affects the waveform and energy distribution of the signal. By analyzing the interference patterns of closely spaced double-layer rebar, their structural characteristics can be effectively identified (Figure 2).

Furthermore, the superposition characteristics of electromagnetic waves are closely related to signal frequency and the physical properties of the propagation medium. Radar waves of different frequencies exhibit distinct propagation behaviors within multilayer rebar structures. In particular, high-frequency and low-frequency signals demonstrate varying reflection and scattering effects. High-frequency signals tend to undergo multiple reflections and scatterings around the target, whereas low-frequency signals penetrate surfaces more effectively. These differences significantly influence the superposition and destructive interference characteristics of the received signals.

Therefore, when detecting double-layer rebar, the superposition characteristics of electromagnetic waves not only affect signal propagation paths and reflection intensity but also play a crucial role in the accuracy of detection results and target identification. By modeling and analyzing the superposition effects of electromagnetic waves, a deeper understanding of multiple reflections, scattering, and interference phenomena can be achieved. This, in turn, enhances the effectiveness of ground-penetrating radar (GPR) and similar technologies in complex media environments.

### 2.3. Forward Modeling for Different Burial Depths

To facilitate the observation and extraction of signal characteristics for single- and double-layer rebar, this study utilizes gprMax 3.0 software [29] to conduct forward modeling simulations for highly reflective media. The specific simulation parameters for concrete structures with high reflectivity are as follows: the vertical spacing of the double-layer reflective medium is set at 30 cm and 40 cm, with a horizontal spacing of 20 cm, and the medium material is concrete. The simulation process employs radar scanning along the y-axis to acquire the necessary data.

**Figure 3:**
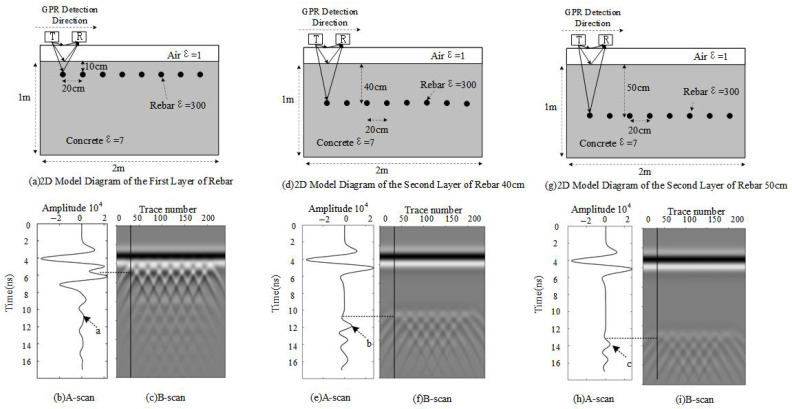
Single-layer rebar model at different burial depths. (**a**) Burial depth of 10 cm. (**b**) A-scan at 10 cm. (**c**) B-scan at 10 cm. (**d**) Burial depth of 40 cm. (**e**) A-scan at 40 cm. (**f**) B-scan at 40 cm. (**g**) Burial depth of 50 cm. (**h**) A-scan at 50 cm. (**i**) B-scan at 50 cm.

Figure 3 illustrates a designed rectangular model containing a single-layer high-reflectivity medium. The model has a length (*L*) of 2 m, a height (*H*) of 1 m, and a width of 0.04 m, which is neglected in the plot. A high-reflectivity medium with a radius of 0.6 mm is distributed within the model, while the remaining part is composed of concrete.

Concrete has a relative permittivity of 6.0, conductivity of 0.01 S/m, and relative permeability of 1.0, which results in moderate electromagnetic wave propagation with weak reflection. In contrast, a high-reflectivity medium with a permittivity of 300, conductivity of $10^{7}$ S/m, and relative permeability of 1.0 reflects waves strongly due to its high conductivity and permittivity.

The simulation employs a GPR antenna with a frequency of 400 MHz, and data acquisition is conducted using a continuous scanning approach.

As observed in Figure 3, regardless of the rebar burial depth, the one-dimensional signal consistently exhibits phase inversion due to the high dielectric constant of the rebar and the negative Fresnel reflection coefficient. In Figure 3b, point a has an amplitude of 0.18, representing a secondary signal generated by the intersection of the first-layer rebar with other reinforcement layers. Similarly, in Figure 3e, point b exhibits an amplitude of 0.69, while in Figure 3h, point c reaches 0.44. These amplitude increases are attributed to the strong reflectivity of rebar as a high-reflectivity medium for electromagnetic waves.

**Figure 4:**
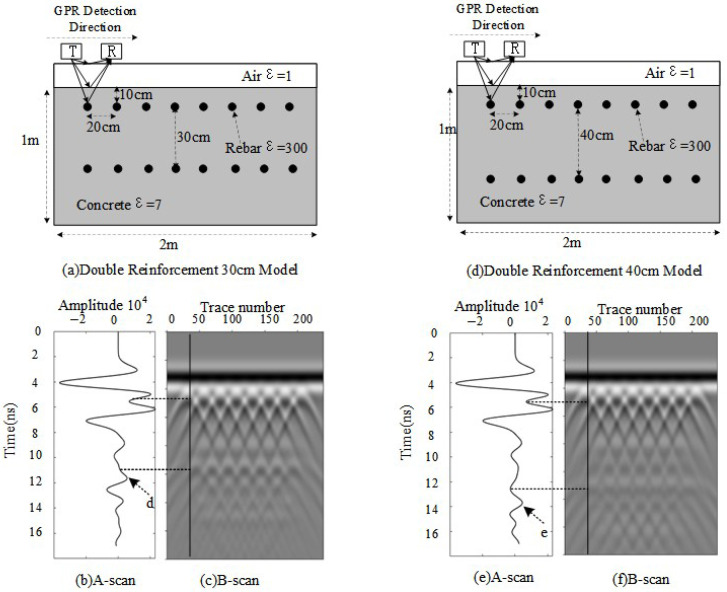
Double-layer rebar model with different spacings. (**a**) Model with 30 cm spacing (**b**) A-scan for 30 cm spacing. (**c**) B-scan for 30 cm spacing. (**d**) Model with 40 cm spacing. (**e**) A-scan for 40 cm spacing. (**f**) B-scan for 40 cm spacing.

When a double-layer rebar configuration is introduced, variations in rebar spacing significantly impact the received signal. To analyze this effect, forward modeling simulations are conducted with rebar spacings of 30 cm and 40 cm, while keeping all other parameters unchanged, as illustrated in Figure 4.

When the spacing between the two layers of rebar is 30 cm, the calculated phase difference is Δϕ≈4.55π, resulting in partial destructive interference and reduced signal amplitude. In Figure 4b, the reflection peak at the position of the second layer of rebar is about 0.55. Compared with the single-layer case, the presence of double layers causes signal attenuation due to interference, though the peak remains positive and clearly visible. When the spacing increases to 40 cm, Δϕ≈6π, corresponding to constructive interference, which enhances the radar echo. In Figure 4e, the reflection peak of the second layer of rebar reaches approximately 0.45, which is significantly higher than that of a single-layer rebar at the same depth, indicating improved detectability due to signal reinforcement.

Thus, regardless of whether constructive or destructive interference occurs, the reflection signals from highly reflective double-layered media consistently exhibit an increase in positive amplitude. Additionally, due to the inherent physical properties of rebar, phase inversion occurs in the reflected signals.

## 3. Multi-Domain Feature Analysis of Aliased Signals

This section further analyzes the differences in phase, amplitude, and frequency characteristics between aliased signals from single- and double-layer rebar. The focus is primarily on time-domain and time–frequency domain feature analysis to better define the distinct signal characteristics of aliased signals in single- and double-layer rebar structures.

### 3.1. Enhanced Characteristics of Normalized Aliased Signal Amplitude in the Time Domain

When using a 400 MHz frequency for simulations based on the forward modeling results, the electromagnetic response of the double-layer rebar structure becomes less distinct due to interference and aliasing effects, leading to constructive interference that affects the interpretation of ground-penetrating radar (GPR) data. The purpose of normalization is to eliminate amplitude differences between signals, ensuring that all signals can be compared on the same scale. The normalization formula is expressed as follows:

(11) $$x_{\mathrm{norm}}=2\cdot \left(\frac{x-\min(x)}{\max(x)-\min(x)}\right)-1$$

In the normalization formula, min(x) represents the minimum value, and max(x) represents the maximum value. The normalized signal $x_{\mathrm{norm}}$ is used to analyze phase variations by comparing the normalized amplitude differences between single-layer and double-layer rebar signals. This process determines whether there are significant amplitude variations in the region of interest.

**Figure 5:**
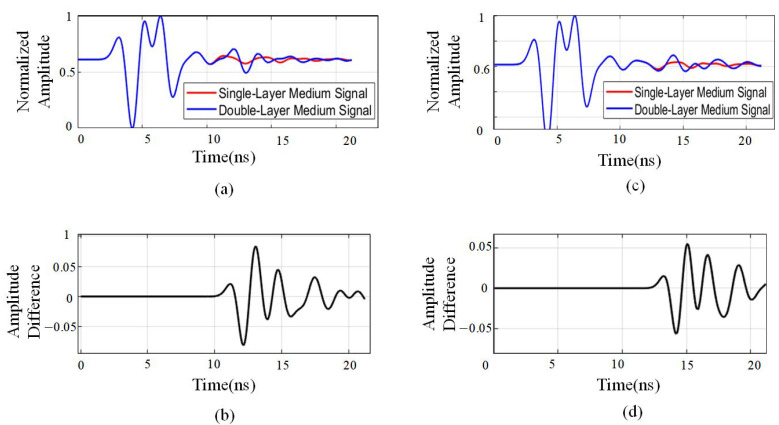
Normalized amplitude difference of signal traces in single- and double-layer concrete structures at 400 MHz low-frequency. (**a**) Normalized amplitude map of double-layer rebar with 30 cm spacing. (**b**) Normalized amplitude difference between double-layer rebar (30 cm spacing) and single-layer rebar. (**c**) Normalized amplitude map of double-layer rebar with 40 cm spacing. (**d**) Normalized amplitude difference between double-layer rebar (30 cm spacing) and single-layer rebar.

The computed value quantifies the relative amplitude difference between the single-layer and double-layer rebar signals, effectively highlighting intensity variations in the time domain. This facilitates the identification of regions where the signals exhibit significant differences, thereby aiding in the characterization of single- and double-layer rebar features. To visualize amplitude differences, the forward modeling simulation data are utilized to normalize the aliased signals of single- and double-layer rebar. For destructive interference, a comparison is made between a single-layer rebar at a burial depth of 10 cm and a double-layer rebar with a 30 cm spacing, as shown in Figure 5a,b. For constructive interference, a comparison is made between a single-layer rebar at a burial depth of 10 cm and a double-layer rebar with a 40 cm spacing, as shown in Figure 5c,d.

The normalized amplitude difference ranges between 0.5 and −0.5, and in certain cases, the two-way travel time exceeds 0.05. This indicates that during the 10–16 ns time window, the signal amplitude difference between single-layer and double-layer media is particularly pronounced. Notably, when encountering medium transitions, the fluctuations in signal intensity increase significantly. This phenomenon can be attributed to multiple reflections, refractions, and attenuation occurring as electromagnetic waves propagate through medium boundaries. These interactions lead to temporal signal dispersion and waveform distortion, especially in regions where the medium transitions are more complex. In such areas, the amplitude and waveform variations are more prominent, highlighting the influence of the medium on signal behavior.

**Figure 6:**
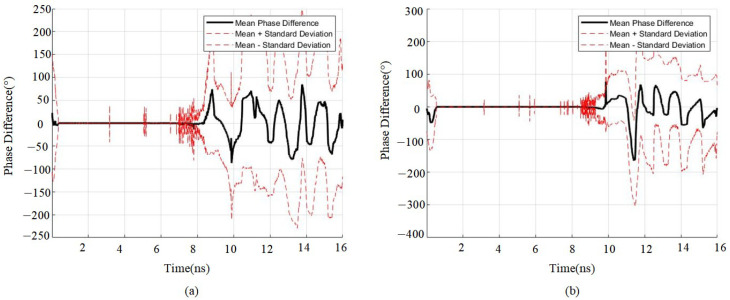
Phase difference between double-layer and single-layer reinforcement; (**a**) 30 cm spacing phase difference between double-layer and single-layer reinforcement; (**b**) 40 cm spacing phase difference between double-layer and single-layer reinforcement.

**Figure 7:**
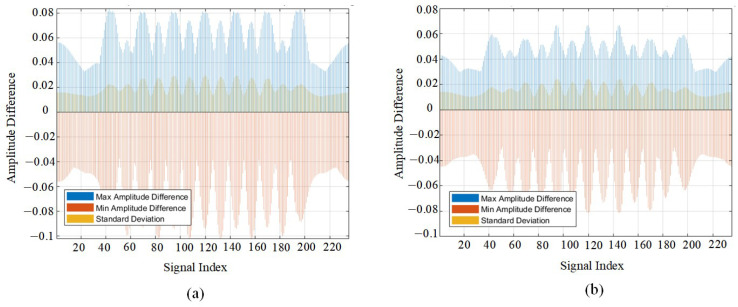
Statistical analysis of amplitude variations in one-dimensional signals for single- and double-layer media. (**a**) Amplitude difference between 30 cm spaced double-layer rebar and single-layer rebar. (**b**) Amplitude difference between 40 cm spaced double-layer rebar and single-layer rebar.

The normalization difference confirms a variation in the amplitude of the phase due to the presence of a second medium layer. Figure 6 shows the statistical differences in phase changes between signals from single and double medium layers. It can be seen that the presence of the double layer of reinforcement causes an increase in the phase change compared to the phase change in the single layer of reinforcing steel. Figure 7 shows the statistical variation in signal amplitude differences for a single double-layer medium. It can be observed that a peak point in the amplitude coincides with the location of the double-layer medium. When comparing the differences between single- and double-layer media, the amplitudes—both positive and negative—of the signal after passing through the double-layer medium increase significantly.

Overall, whether the scanning signal contains a medium or not, the double-layer medium has a significant impact on signal amplitude within the 8–16 ns two-way travel time range. When electromagnetic waves encounter medium interfaces, they undergo reflection, refraction, and attenuation, leading to variations in signal intensity, waveform distortion, and temporal dispersion. These transformations provide critical insights for further medium characterization and confirm that amplitude growth is not solely limited to constructive or destructive interference.

### 3.2. Time–Frequency Characteristic Analysis of Phase-Inverted Reflections Using the Hilbert Transform

A one-dimensional signal (A-scan) represents the reflected waveform of radar waves at a single detection point. The electromagnetic properties of different media influence the phase shift of radar signals, particularly in the presence of double-layer rebar (such as reinforcement bars or metal pipes). Phase characteristics can be utilized to identify the structure and layering of the target. Due to the high dielectric constant of double-layer rebar, when an electromagnetic wave reaches the medium, the reflection coefficient becomes negative, resulting in phase inversion relative to the incident wave, as described by Equation (4). To quantitatively analyze phase variations, we employ the Hilbert transform to extract instantaneous phase and compare phase characteristics to assess the impact of single-layer and double-layer rebar structures.

In radar signal processing, the Hilbert transform is utilized to enhance the characteristics of reflected waves and analyze signal variations at different medium interfaces. For a real signal s(t), the Hilbert transform is defined as follows:

(12) $$S_{a}\left(t\right)=s\left(t\right)+iH\left[s(t)\right]$$

The primary function of the Hilbert transform is to extend a real signal into its complex analytic signal $S_{a}\left(t\right)$, which is defined as follows:

(13) $$H\left[s(t)\right]=\frac{1}{\pi }\int_{-\infty }^{\infty }\frac{s(\tau )}{t-\tau }d\tau$$

In this equation, s(t) represents the original signal, H[s(t)] denotes the Hilbert-transformed signal, and *i* is the imaginary unit. The analytic signal can be utilized to extract key signal attributes such as instantaneous amplitude, instantaneous phase, and instantaneous frequency.

The core function of the Hilbert transform is to apply a $-90^{\circ }$ phase shift to the negative frequency components and a $+90^{\circ }$ phase shift to the positive frequency components. Consequently, in the frequency domain, the Hilbert transform can be expressed as follows:

(14) $$H{\left[s(t)\right]}^{F}⟷i\;\mathrm{sgn}\left(f\right)S\left(f\right)$$

(15) $$\mathrm{sgn}\left(f\right)=\left\{\begin{array}{cc} 1, & f>0\\ 0, & f=0\\ -1, & f<0 \end{array}\right.$$

In this equation, S(f) represents the Fourier transform of the original signal, and sgn(f) is the sign function. This property enables the Hilbert transform to construct analytic signals, making it a powerful tool for envelope analysis, instantaneous frequency analysis, and other signal processing applications.

**Figure 8:**
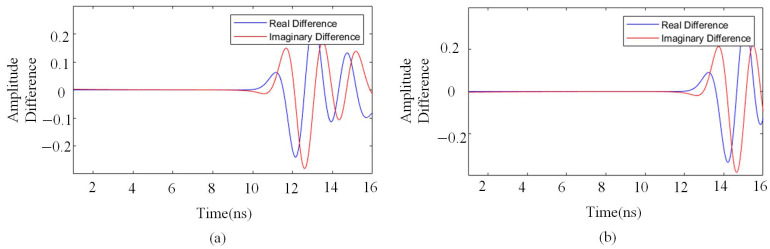
Differences in real and imaginary parts of single- and double-layer medium signals with different spacings; (**a**) 30 cm spacing; (**b**) 40 cm spacing.

By applying the Hilbert transform to the signal, both its envelope and phase characteristics can be analyzed. The real part typically represents the primary waveform of the signal. By comparing the real parts of two sets of signals, their structural differences can be observed. As shown in Figure 8a,b, the real parts of single-layer and double-layer signals exhibit significant differences within the 10–16 ns range. This suggests variations in signal propagation characteristics over time, which may be associated with differences in structure or medium propagation properties.

The imaginary part provides insights into phase variations, particularly in detecting time shifts such as signal delays or advancements. Comparing the imaginary parts of single-layer and double-layer medium signals, the difference map reveals significant phase variations, indicating phase delays or advancements in the double-layer medium signal propagation. Although discrepancies exist between the real and imaginary parts of the figures, these differences fundamentally result from the combined effects of amplitude and phase variations.

**Figure 9:**
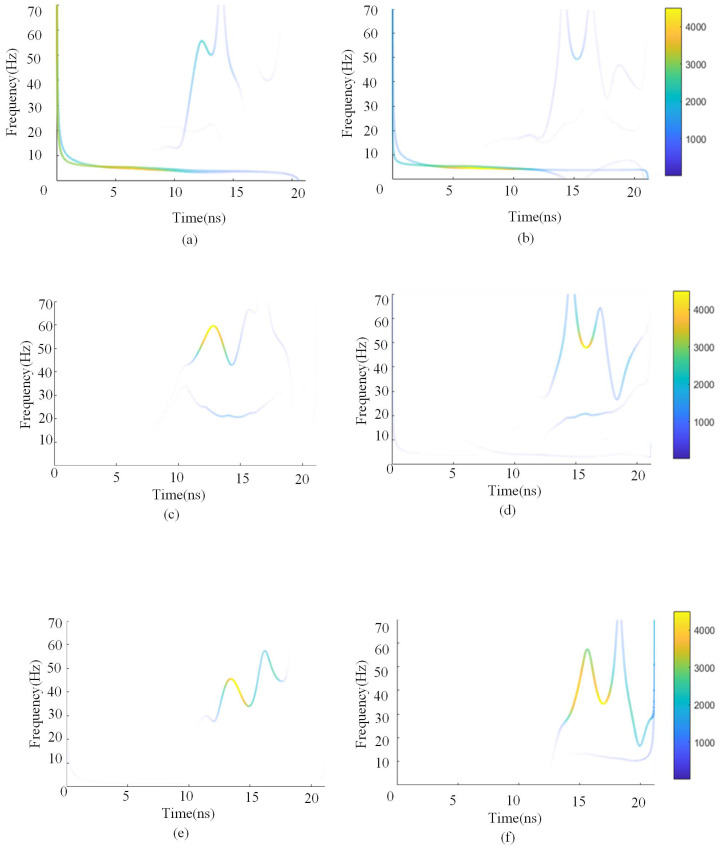
Hilbert spectrum differences between double-layer medium signals and single-layer rebar signals. (**a**) Distance 30 cm apart, 45th radar trace. (**b**) Distance 40 cm apart, 45th radar trace. (**c**) Distance 30 cm apart, 90th radar trace. (**d**) Distance 40 cm apart, 90th radar trace. (**e**) Distance 30 cm apart, 160th radar trace. (**f**) Distance 40 cm apart, 160th radar trace.

Figure 9 illustrates the differences in Hilbert spectra between single-layer and double-layer media, revealing the distribution of instantaneous frequency in the signals. Variations in frequency may reflect phase shifts during signal propagation or differences in propagation media. Both subfigures exhibit strong energy concentration in the low-frequency band, indicating that the primary reflected energy is concentrated in the lower frequencies—a typical characteristic of radar wave propagation in media such as concrete. Compared to Figure 9a, the mid-frequency components in Figure 9b appear more dispersed and show faster energy attenuation, likely due to changes in reflection paths caused by different rebar spacings in the double-layer structure. In the difference spectrum, the main energy is concentrated within 0–10 Hz, and local high-frequency peaks appear in the 10–20 ns range, indicating a periodic interference pattern caused by the double-layer rebar structure. On this basis, we further incorporated multiple sets of simulation data—including various rebar spacings and burial depths for both single- and double-layer configurations—to compare their Hilbert spectrum differences and enhance the robustness and generalizability of the results, as shown in Figure 9c–f. These additional results further validate the effectiveness of the Hilbert spectrum in identifying complex overlapping signals.

Over time, the instantaneous frequency of the signal gradually increases from the low-frequency region, reaches a peak, and then begins to decline. This variation may reflect specific characteristics of signal propagation. The instantaneous frequency range, approximately 0 Hz to 70 Hz, indicates a relatively narrow frequency distribution, with significant frequency fluctuations occurring within a short time window.

By comparing the instantaneous amplitude of single-layer and double-layer medium signals (i.e., the amplitude of the Hilbert spectrum), their energy differences in the time domain can be observed. Additionally, the real and imaginary part differences obtained from the Hilbert transform further illustrate the effects of multiple phase inversions, indicating that radar waves undergo multiple reflections and interferences between rebars. This results in periodic phase variations, confirming that double-layer rebar signals exhibit phase reversals at later time intervals.

Between 10 ns and 16 ns, the influence of multiple reflections and propagation effects on double-layer rebar signals becomes more pronounced. The phase variations in the time domain become increasingly significant, and the signal complexity in the frequency domain intensifies, leading to differences in frequency energy distribution. Consequently, double-layer rebar signals exhibit a more intricate and enriched frequency spectrum compared to single-layer signals.

### 3.3. Analysis of STFT Spectrum Differences and Reflection Complexity in Aliased Signals

The short-time Fourier transform (STFT) is employed for time–frequency analysis of these signals, exploring differences in reflection intensity and phase to provide valuable insights for signal identification and classification.

STFT is a time–frequency analysis method that applies a localized Fourier transform using a sliding time window. It enables the extraction of frequency components at different time instances, making it particularly effective for analyzing non-stationary signals. In ground-penetrating radar (GPR) signal analysis, STFT helps reveal signal variations in both the time and frequency domains, especially in scenarios involving multi-layer media and aliased reflections.

The mathematical expression for STFT is given as [30]:

(16) $$X\left(t,f\right)=\int_{-\infty }^{\infty }x\left(\tau \right)\cdot w\left(\tau -t\right)\cdot e^{-j2\pi f\tau }d\tau$$

In this equation, x(τ) represents the original signal, w(τ−t) is the window function, and X(t,f) denotes the time–frequency representation.

By selecting signals containing double-layer rebar for comparison, we analyze the original A-scan signals from the same column for both single-layer and double-layer media. In the time domain, the waveform of double-layer rebar signals appears more complex than that of single-layer rebar signals, indicating the presence of additional reflection components. This suggests that reflections induced by double-layer rebar structures are inherently more intricate, leading to greater amplitude variations and incorporating multiple secondary reflections within the signal.

**Figure 10:**
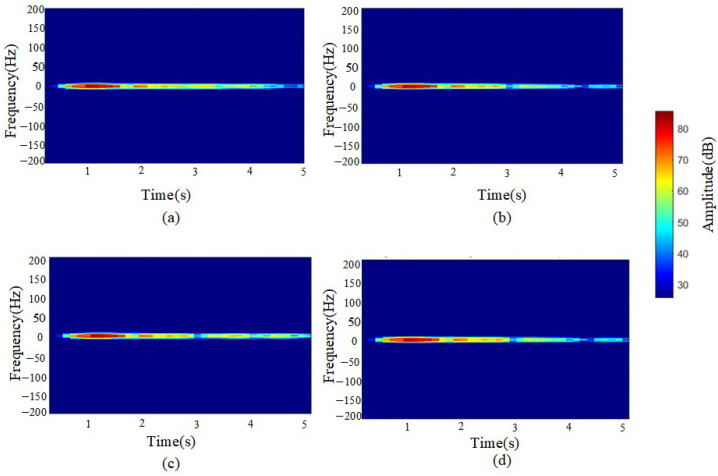
STFT time–frequency 2D transformation maps (**a**) Double-layer rebar with 30 cm spacing. (**b**) Single-layer rebar (**c**) Double-layer rebar with 40 cm spacing. (**d**) Single-layer rebar.

Figure 10 reveals subtle differences between single-layer and double-layer media in the frequency domain. The double-layer medium exhibits a broader spectral range within the 3–4 s time window, with more widely distributed signal energy, indicating a stronger impact of the double-layer medium on reflections across different frequencies. The energy amplitude in the 3–5 s range reaches approximately 50–60.

In contrast, the single-layer medium demonstrates a more concentrated spectral distribution, with energy primarily confined to lower frequencies. Its reflected signals exhibit a relatively stable spectral pattern in the time–frequency diagram, with frequency components concentrated in the lower frequency band and minimal variation. The energy amplitude in the 3–5 s range is approximately 35–50.

This indicates that the reflection signals of the single-layer medium are relatively simple, with a more stable reflection pattern and minimal frequency variation. In the STFT time–frequency diagram, the double-layer medium exhibits multiple concentrated energy regions, with a broader energy distribution, suggesting the presence of energy superposition or interference effects.

To clearly observe the differences between single-layer and double-layer media in GPR signals, a differential short-time Fourier transform (STFT) analysis was performed, as shown in Figure 10.

**Figure 11:**
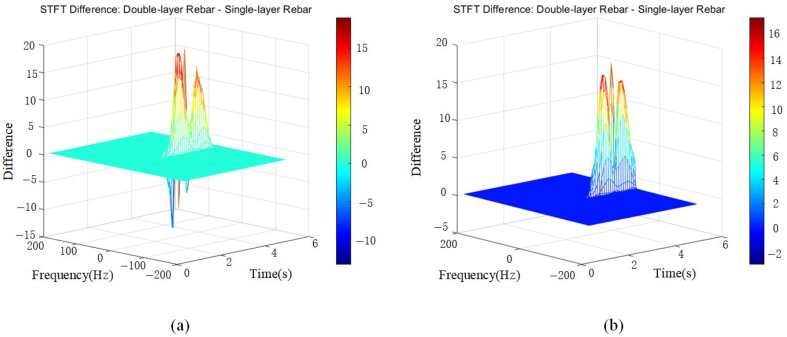
A 3D difference map of STFT transformations for single- and double-layer media. (**a**) Double-layer rebar with 30 cm spacing. (**b**) Double-layer rebar with 40 cm spacing.

Significant differences exist between single-layer and double-layer medium signals, likely due to the distinct propagation characteristics of double-layer media, such as multiple reflections and multipath propagation. In Figure 11, the later-stage frequency difference falls within the 0–20 range, exhibiting varied energy distributions. Regarding amplitude differences in the time-domain signals, high-amplitude regions in the STFT difference map suggest signal energy enhancement, indicating that the rebar spacing aligns with the wavelength, leading to constructive interference. Aliased signals may exhibit higher amplitudes than the original signals, suggesting that within specific time and frequency ranges, the amplitude differences between single-layer and double-layer media signals are significantly pronounced.

Through STFT transformation and comparative analysis, it is observed that double-layer medium signals exhibit a broader energy distribution and greater frequency variations, indicating a more complex reflection pattern.

**Figure 12:**
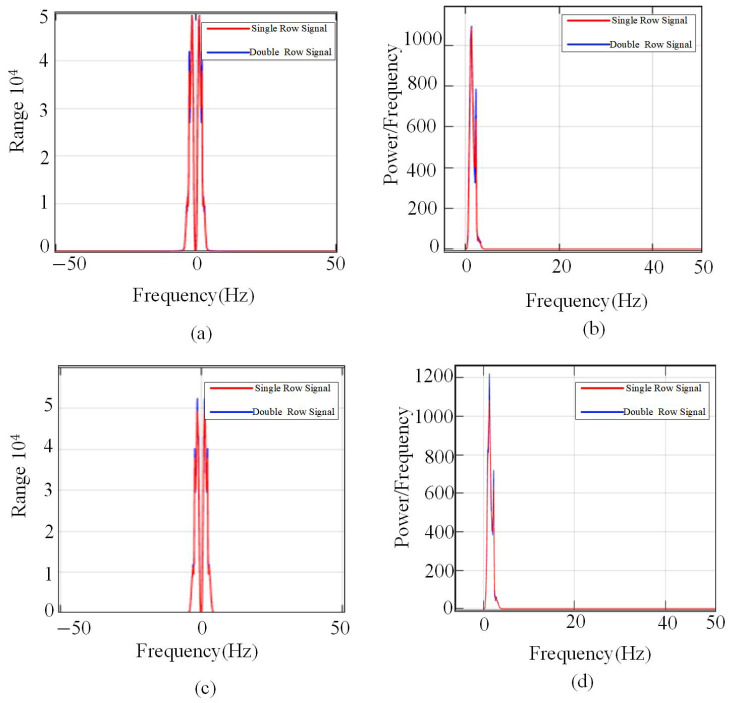
Difference map of FFT transformations for single- and double-layer media. (**a**) Bilateral spectrograms with double reinforcement spaced 30 cm apart. (**b**) Power Spectrogram of Double Layer Rebar Spacing 30 cm. (**c**) Bilateral spectrogram of double-layer reinforcement spacing 40 cm. (**d**) Power Spectrum of Double Layer Bar Spacing 40 cm.

The method has a higher time–frequency resolution capability compared with the traditional Fourier transform. In the traditional Fourier transform, as shown in Figure 12, the overall frequency components and energy distribution of the signal show a certain degree of overlap and blurring, making it difficult to clearly distinguish the differences between different signal features. Since the Fourier transform converts time domain signals to frequency domain signals to provide frequency domain information, it has limitations in dealing with non-smooth signals or local feature identification. In Figure 12, it can be observed that the spectral curves of the original signal and the comparison signal are relatively close to each other, and the difference in energy distribution is not obvious, which makes it difficult to use as an effective basis for accurate identification or classification. In contrast, for example, the short-time Fourier transform not only retains the frequency domain information but also takes into account the time locality, making the detection of key features in the signal more accurate and reliable.

Based on subjective evaluation and experimental conclusions, the classification criteria are determined by analyzing phase, amplitude, and amplitude variation in time-series signals. Firstly, when electromagnetic waves propagate from the normal lining structure to the rebar, if the signal does not reach the rebar, the waveform undergoes exponential attenuation. This phenomenon is closely related to the propagation characteristics of electromagnetic waves in different media. By examining the A-scan waveform transformation caused by the physical property differences between double-layer rebar and low-reflectivity media, and incorporating frequency variations, the classification criteria are summarized as follows:

Based on the above-mentioned classification criteria, the cross-reflected wave from the first-layer medium combines with the reflected wave from the second-layer medium to produce a greater amplitude, resulting in an increase in the peak value of the reflected signal, which manifests as amplitude variations.

Furthermore, when electromagnetic waves propagate from low-permittivity concrete to high-permittivity rebar, the reflection coefficient becomes negative, leading to a $180^{\circ }$ phase inversion. This phase shift influences both negative and positive peak points of the signal and contributes to increased frequency energy in the presence of a double-layer medium.

**Figure 13:**
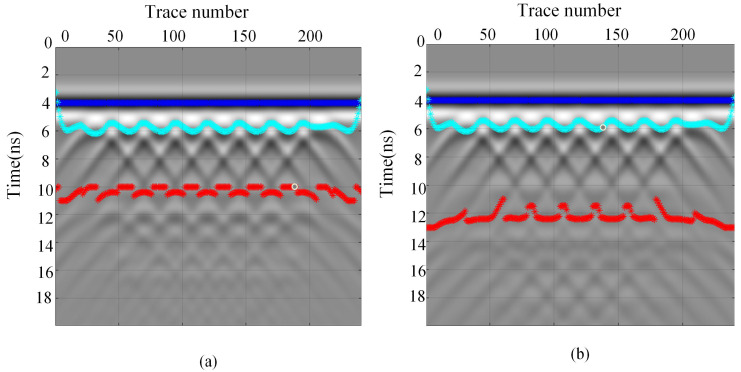
Simulation-based identification of double-layer rebar with different spacings. (**a**) 30 cm spacing. (**b**) 40 cm spacing.

These findings indicate that phase and amplitude differences serve as critical indicators for identifying double-layer media, providing a more precise method for detecting double-layer rebar. These phenomena indicate that phase differences and amplitude variations become important criteria for identifying double-layer media, providing a more accurate method for determining the positions of double-layer rebars. Based on these features, the double-layer rebars are labeled in the simulation data, as shown by the light blue curve in Figure 13. The first-layer rebar forms a complete hyperbolic curve due to the integrity of the signal. In contrast, the second-layer rebar exhibits an incomplete hyperbolic curve due to signal aliasing, as shown by the red curve in Figure 13. Furthermore, the deeper the burial depth of the second-layer rebar, the more severe the signal aliasing becomes, resulting in discontinuities in the hyperbolic curve.

## 4. Application of Aliased Signal Recognition in Tunnel Rebar Detection

**Figure 14:**
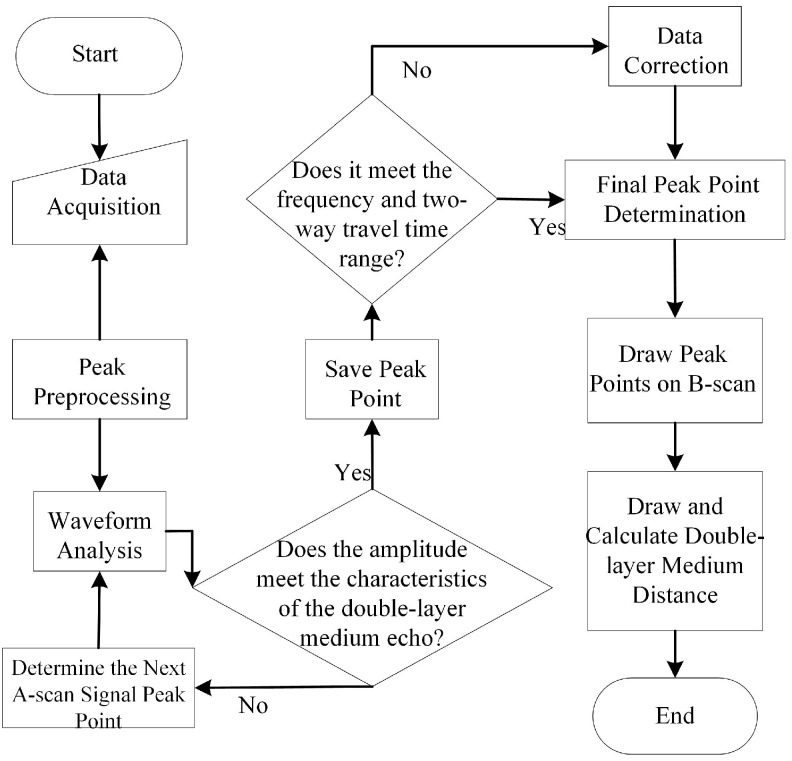
Workflow of the peak extraction method.

Based on the aforementioned characteristics, the aliased signal peak extraction method is employed, following the workflow outlined in Figure 14. When analyzing A-scan data, the reflection points of double-layer rebar are first compared. Subsequently, in the B-scan image, the apex points of the hyperbolas corresponding to the first and second layers of rebar are identified and marked. By comparing the positions and shapes of different reflection points, the two layers of rebar can be effectively distinguished.

The Husa Tunnel, located in western Yunnan Province, has an elevation ranging from 1404 m to 2020 m and is the longest tunnel along the Tengchong–Longchuan Expressway, classified as an extra-long tunnel. At the right-side entrance of the Husa Tunnel (Tengchong side), spanning YK81 + 305 to YK81 + 630, tunnel inspection requirements dictate that seven survey lines are arranged along the tunnel’s longitudinal direction, with a 2 m spacing between measurement lines, centered around the tunnel crown.

**Figure 15:**
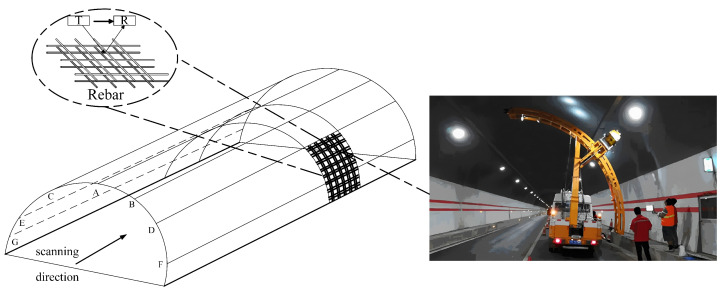
Husa Tunnel Inspection Diagram.

As shown in Figure 15, a ground-penetrating radar (GPR) system is mounted on the tunnel scanning vehicle to conduct tunnel inspections. The transmitter (T) emits electromagnetic waves, while the receiver (R) captures the reflected signals. During construction, the double-layer rebar spacing within the reinforcement mesh of the tunnel lining ranges approximately between 25 cm and 30 cm. The tunnel lining inspection is carried out using a GSSI SIR-3000 (GSSI, Nashua, NH, USA) system equipped with a GSSI shielded 400 MHz antenna.

The equipment is equipped with antenna shielding and strong anti-interference capabilities, allowing for large-area detection while providing high-resolution imaging.

During the actual inspection process, the GPR antenna moves along the survey line direction, performing a continuous scan of the right arch waist position of the Husa Tunnel lining structure. The system generates real-time displayed monitoring data and records the results accordingly.

As shown in Table 1, the data were sourced from the Yunnan Aerospace Engineering Geophysical Survey & Testing Co., Ltd., (located in Kunming, China) as part of the Yunnan Tengchong–Longchuan Expressway Engineering Report.

In real tunnel lining GPR data, after the first-layer rebar signal is generated, the dielectric constant of the rebar reaches as high as 300. The electromagnetic waves reflected by the rebar undergo additional reflections at the air–concrete interface, then continue propagating and are further reflected at the rebar-concrete boundary.

**Figure 16:**
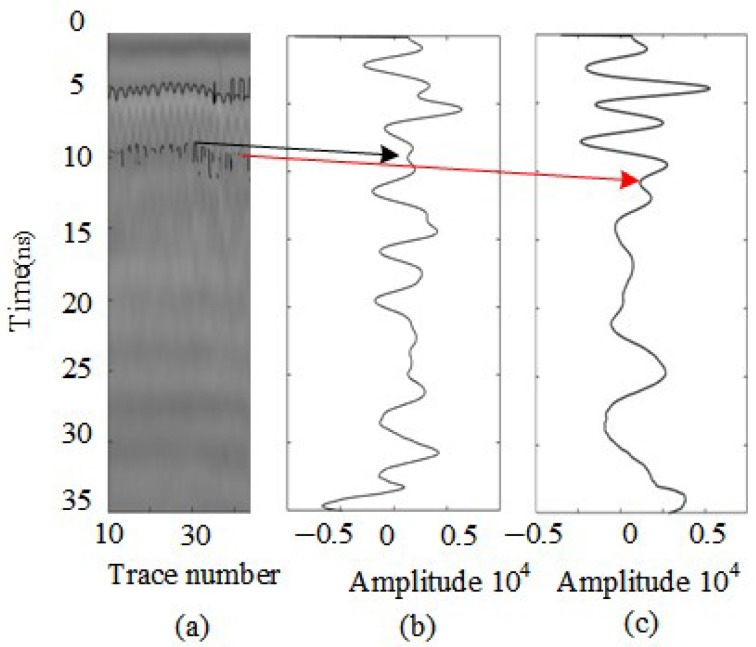
Initial identification of double-layer rebar using real data. (**a**) B-scan image (**b**) A-scan image with rebar feature. (**c**) A-scan image without rebar features. Red arrows are unidentified signal feature locations. Black arrows are identified signal feature locations.

As shown in Figure 16, the echo from the second-layer rebar exhibits similar phase and amplitude variations to the first-layer rebar. The presence of rebar is characterized by an amplitude lower than the adjacent in-phase negative peak, while horizontal signal aliasing leads to signal intersections around 10 ns, as indicated by the black line in Figure 16. This behavior aligns with the time-domain characteristics extracted from the signal processing analysis.

Due to the consistent phase variations between the aliased signals and the second-layer rebar signals, signal superposition occurs, leading to amplitude enhancement. Around 11 ns, the amplitude weakens, preventing the signal from decaying at a uniform rate. This may be caused by excessive coherent interference between signals, which can result in signal feature loss, as shown at the reflection point indicated by the red line in Figure 16.

**Figure 17:**
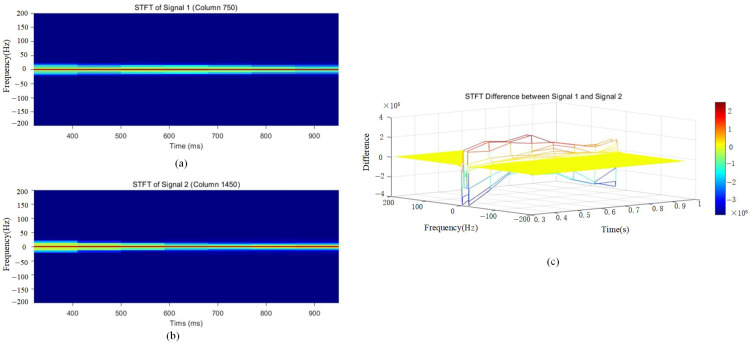
STFT comparison diagram. (**a**) Time–frequency STFT map at the identified point. (**b**) Time–frequency STFT map at unidentified points. (**c**) Three-dimensional (3D) time–frequency difference map.

**Figure 18:**
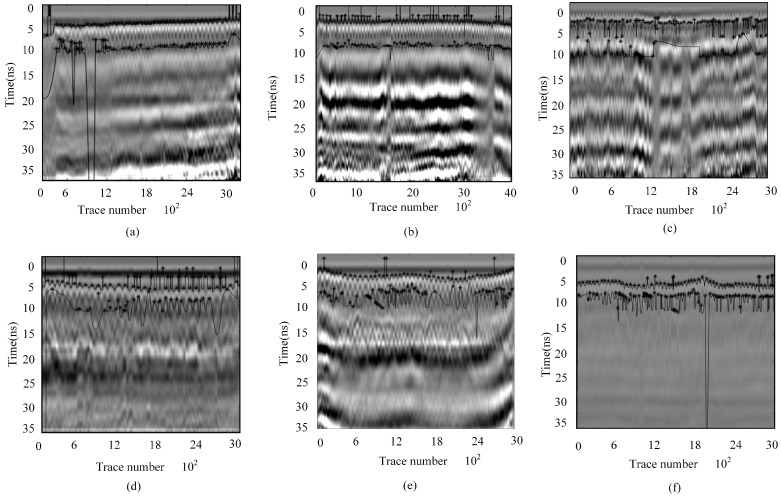
Identification result diagram. (**a**) E-YK81 + 435 + 310 Survey Line 1. (**b**) C-YK81 + 500 + 310 Survey Line 2. (**c**) A-YK81 + 365 + 316 Survey Line 1. (**d**) E-YK81 + 610 + 500 Survey Line 1. (**e**) E-YK81 + 610 + 500 Survey Line 2. (**f**) D-YK81+ 626.5 + 720 Survey Line 1.

The data are preprocessed, and a peak extraction algorithm is applied in the time domain. Using frequency energy differences in the time–frequency domain, signals are filtered, and the short-time Fourier transform (STFT) is employed to compare frequency-domain reflection intensities for two different cases, as shown in Figure 17. By analyzing the phase variations in the processed one-dimensional signal, the negative peak locations associated with rebar presence are identified. Amplitude variations are further examined to determine the negative and positive peak points, using abnormal amplitude changes relative to adjacent peaks as constraint conditions. Finally, the data are reconstructed, and the final rebar identification results are presented in Figure 18.

Several segments along the tunnel alignment are randomly selected for comparison with the engineering experiment report. The distance calculation formula is used to determine the burial depth (*h*) of the rebar, which can be expressed as follows:

(17) $$h=\frac{v(t_{r}-t_{0})}{2}$$

Given the propagation time of the electromagnetic wave from concrete to the first-layer rebar ($t_{0}$) and the propagation time to the second-layer rebar ($t_{r}$), the dielectric constant of reinforced concrete structures is typically relatively high, while conductivity becomes a key factor influencing electromagnetic wave propagation characteristics.

Therefore, the propagation speed of electromagnetic waves in a high-reflectivity medium can be characterized using the dielectric constant, expressed as follows:

(18) $$v=\frac{c}{\sqrt{\varepsilon }}$$

In this equation, *c* represents the speed of light, and ε denotes the dielectric constant of the medium through which the wave propagates. By applying this formula, the spacing between the two layers of rebar is determined. The calculated results are presented in Table 2.

In the identification method, each A-scan can be utilized to determine thickness. Compared to manual identification, automated identification not only provides an average vertical spacing that can be cross-verified with sampled values, but also calculates the relative error in the vertical spacing of double-layer rebar within the lining. As detailed in Table 2, the sampling relative error is predominantly controlled within the range of 0.03% to 5.66%, ensuring that the deviation of the rebar mesh remains within the permissible tolerance limits.

## 5. Discussion and Conclusions

This study analyzes coherent constructive interference caused by electromagnetic wave superposition and develops a feature-based aliased signal peak extraction algorithm. The algorithm continuously filters specific peak points, mapping them onto a 2D representation. By computing the time difference between peak points and incorporating the signal propagation speed, the thickness of the double-layer high-reflectivity medium can be accurately determined. The key conclusions are as follows:

In the time domain, apart from phase variations caused by dielectric constants and amplitude increases due to signal aliasing, the time–frequency domain analysis reveals that the frequency energy of a double-layer rebar is 20% higher than that of a single-layer rebar. Using the feature-based peak extraction method, data labeling was conducted to enhance identification accuracy. The algorithm was applied in the Husa Tunnel, located in western Yunnan, for the identification of double-layer rebar using radar detection data. A comprehensive analysis was conducted in both the time domain and time–frequency domain to evaluate the signal characteristics.

The calculated results indicate that the spacing between the double-layer rebar predominantly falls within the range of 25 cm to 40 cm, while the relative position of the first-layer rebar remains stable, typically between 14 cm and 20 cm.

A sampling comparison was performed between the detection results and the actual engineering report. The relative errors of most sampled data were well controlled within the range of 0.03% to 4.25%, ensuring compliance with engineering inspection standards. However, some errors exceeded 5%, which could be attributed to data fluctuations and environmental measurement conditions.

Currently, the feature extraction algorithm based on double-layer rebar signal characteristics is primarily applied to the inspection of intact tunnel linings. However, during actual tunnel operation, various structural challenges such as water seepage and voids may arise, which can significantly alter the GPR-detected signal characteristics.

In light of this, future research will focus on analyzing the impacts of different tunnel defects on signal characteristics, aiming to develop a feature extraction algorithm adaptable to complex operating conditions. This advancement will enhance the algorithm’s effectiveness across diverse tunnel environments, providing a more robust technical foundation for tunnel safety assessment and maintenance management.

## Figures and Tables

**Table 1:** Support parameters of composite lining at the right tunnel entrance of the Husa Tunnel.

Mileage	Rock Class	Preliminary Design			Changes		
		Thickness (cm)	Strength (MPa)	Rebar Distance (cm)	Thickness (cm)	Strength (MPa)	Rebar Distance (cm)
YK81 + 310.54∼358	$V_{2}$	60	30	20	-	-	30
YK81 + 358∼398	$V_{2}$	50	30	20	50	30	40
YK81 + 389∼434	$V_{2}$	50	30	20	45	30	30
YK81 + 434∼506	$V_{1}$	50	30	20	40	30	40
YK81 + 506∼640	$V_{1}$	50	30	24	45	30	25

**Table 2:** Husa Tunnel secondary lining double reinforcement spacing table.

Mileage	Survey Points	Survey Line	Vertical Spacing (m)		Relative Error (%)
			Designed	Measured	
YK81 + 365∼345	3000∼6000	A	0.3	0.328	9.33%
YK81 + 630∼610	5500∼7500	B	0.3	0.317	5.66%
YK81 + 615∼500	1732∼2931	B	0.25	0.257	2.80%
YK81 + 615∼500	20,000 ∼23,000	C	0.3	0.290	3.00%
YK81 + 435∼410	24,000∼26,600	E	0.3	0.306	2.00%
YK81 + 435∼310	2000∼14,400	E	0.4	0.409	2.25%
YK81 + 615∼500	8086∼11,160	E	0.3	0.310	3.33%

## Data Availability

The data used to support the findings of this study are available from the corresponding author upon request.
